# Immunoinhibitory effects of anti-tuberculosis therapy induce the host vulnerability to tuberculosis recurrence

**DOI:** 10.1128/spectrum.00412-24

**Published:** 2024-05-29

**Authors:** Isha Pahuja, Antara Ghoshal, Ahmed Abdallah Okieh, Akanksha Verma, Kriti Negi, Meetu Agarwal, Nidhi Subhash Chandra, Saurabh Kumar Sharma, Ashima Bhaskar, Ved Prakash Dwivedi

**Affiliations:** 1 Immunobiology Group, International Centre for Genetic Engineering and Biotechnology, New Delhi, India; 2 Department of Molecular Medicine, Jamia Hamdard University, New Delhi, India; 3 Department of Microbiology, Ram Lal Anand College, University of Delhi, New Delhi, India; 4 School of Computer & Systems Sciences, Jawaharlal Nehru University, New Delhi, India; Foundation for Innovative New Diagnostics, Geneve, Switzerland

**Keywords:** Tuberculosis, DOTS, drugs, recurrence, T cell memory

## Abstract

The host immune responses play a pivotal role in the establishment of long-term memory responses, which effectively aids in infection clearance. However, the prevailing anti-tuberculosis therapy, while aiming to combat tuberculosis (TB), also debilitates innate and adaptive immune components of the host. In this study, we explored how the front-line anti-TB drugs impact the host immune cells by modulating multiple signaling pathways and subsequently leading to disease relapse. Administration of these drugs led to a reduction in innate immune activation and also the cytokines required to trigger protective T cell responses. Moreover, these drugs led to activation-induced cell death in the mycobacterial-specific T cell leading to a reduced killing capacity. Furthermore, these drugs stalled the T cell differentiation into memory subsets by modulating the activation of STAT3, STAT4, FOXO1, and NFκB transcription factors and hampering the Th1 and Th17-mediated long-term host protective memory responses. These findings suggest the urgent need to augment directly observed treatment, short-course (DOTS) therapy with immunomodulatory agents to mitigate the adverse effects linked to the treatment.

**IMPORTANCE:**

As a central component of TB eradication initiatives, directly observed treatment, short-course (DOTS) therapy imparts immune-dampening effects during the course of treatment. This approach undermines the host immune system by delaying the activation process and lowering the immune response. In our investigation, we have unveiled the impact of DOTS on specific immune cell populations. Notably, the signaling pathways involving STAT3 and STAT4 critical for memory responses and NFκβ associated with pro-inflammation were substantially declined due to the therapy. Consequently, these drugs exhibit limited effectiveness in preventing recurrence of the disease. These observations highlight the imperative integration of immunomodulators to manage TB infection.

## INTRODUCTION

Although the World Health Organization (WHO) Global Tuberculosis Programme aimed to manage tuberculosis (TB) incidence by 80% by 2030, the intricacies associated with direct observed therapy, short course (DOTS) therapy are making the population highly vulnerable to infection (1). Adverse drug reactions from anti-tubercular therapy (ATT) results in hepatotoxixity and low CD4^+^ counts that further prompt more patient suffering and hospitalization (2). Despite extensive efforts to eliminate TB worldwide, the emergence of drug-resistant TB is a threat to the global population. Drug-resistant TB is a severe manifestation of the disease wherein *Mycobacterium tuberculosis* shows resistance to the most potent first-line drugs, namely, isoniazid (INH) and rifampicin (RIF) (3). Approximately 15% of patients succumb to drug-resistant TB. The overall rise in TB incidence is largely attributed to the impact of COVID-19 on the detection of new TB cases (4). The BPaLM (nedaquiline [B], pretomanid [Pa], linezolid [L[, and moxifloxacin [M]) regimen is administered in the cases of drug-resistant TB and demonstrates success in controlling mortality and preventing treatment failure although it is accompanied by adverse effects (5). In addition to adult pulmonary TB, the impact of ATT among children is devastating in poor and malnutrition regions (6). During the DOTS medication, INH, and RIF form the chief component of ATT as these drugs are consistently given during the whole therapy course. However, it was first reported by Tousif et al*.* that, INH impedes CD4^+^ T cells and makes the host more susceptible to reactivation and reinfection of the disease (7). Moreover, several studies have indicated that the administration of INH during TB therapy is linked to peripheral neuropathies, seizures, hypersensitivity, and hematologic disorders, even when prescribed at standard doses. These adverse effects often compel the patients to discontinue the drug (8). Additionally, the prolonged treatment for more than 6 weeks with RIF expedites suppression in circulating T cells during active TB (9). TB patients taking ethambutol (EMB) or those with ceased therapy for more than 6 months manifested consequential drug toxicity in visual functions and optic neuropathy (10). Pyrazinamide (PYZ), another anti-TB drug crucial for clearing bacteria, is primarily associated with serious adverse events experienced by 10–25% of patients encountering side effects. It is essential to address this situation effectively to prevent the development of drug resistance (11). As mentioned, the drug cocktail given during TB has adverse drug reactions, due to which multiple risk factors such as hepatotoxicity, exanthema, arthralgia, gastrointestinal disorders, allergic reactions, and neurological disorders are correlated among pulmonary TB patients (12, 13). Although many studies reported the toxicity induced by individual drugs, it is crucial to decode the after-effects of the prevailing standard regimen on the host’s innate and adaptive immunity. Upon TB infection, macrophages serve as the first line of defense and induce multiple mechanisms in suppressing *M. tuberculosis*. These cells also act as antigen-presenting cells (APC) that further instigate T-cell activation to subvert the pathogenic prevalence (14). T-cells further expedite the host resistance against TB by differentiating into Th1 and Th17 cell-mediated immune responses to exterminate the active pathogen (15). Yet the impact of DOTS on these cell populations is poorly understood. Thus, we aimed to understand the influence of ATT on innate and adaptive immune arms that modulate long-term memory responses in preventing TB recurrence and reactivation.

We observed that DOTS therapy modulates the mitogen-activated protein kinase (MAPK) signaling pathway and dampens innate immunity. Furthermore, the effect was more prominent in T-cell populations where CD4^+^ and CD8^+^ T-cell activation dramatically decreased after the treatment with these antibiotics. Moreover, we also observed activation-induced cell death (AICD) on both CD4^+^ and CD8^+^ T cells when treated with INH. We further monitored the killing potential of these *M. tuberculosis* primed T cells after antibiotics treatment and observed a noteworthy reduction in the T-cell’s ability to effectively eliminate the bacteria. Interestingly, antibiotic treatment altered the T-cell immune signaling and affected the generation of memory T-cells, which are required for long-term protection against TB. In agreement with the previous studies, we also observed an increase in the TB recurrence rate as a result of reduced long-term memory cells.

## RESULTS

### DOTS treatment curtails intracellular bacteria and macrophage activation upon *M. tuberculosis* infection

**FIG 1:**
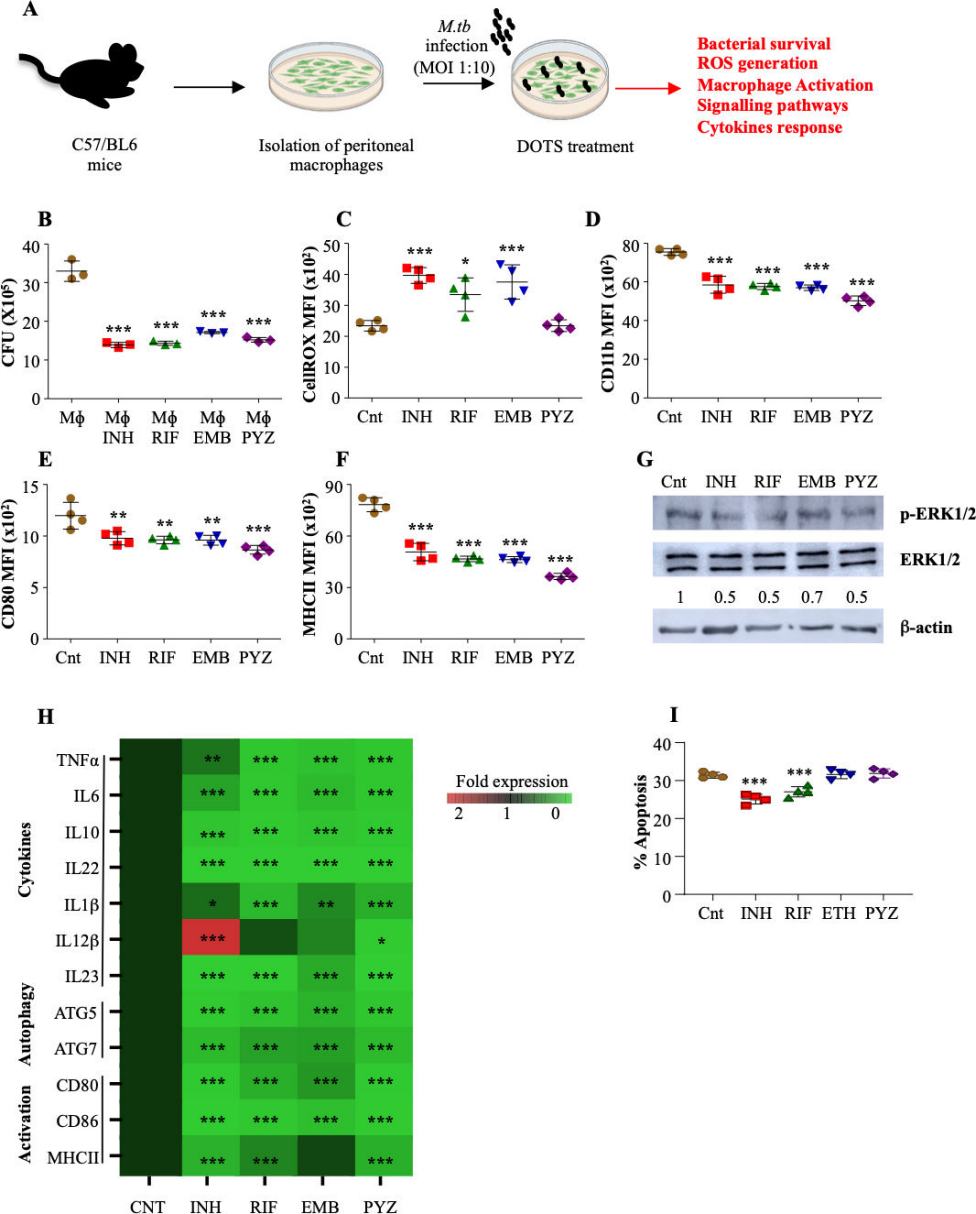
DOTS treatment negatively regulates the macrophage defense mechanisms against *M. tuberculosis*. (A) Schematic representation of the macrophage infection model. Peritoneal macrophages isolated from C57BL6 mice were infected with *M. tuberculosis* H37Rv at a multiplicity of infection (MOI) of 1:10 followed by treatment with 1 µg/mL of INH, RIF, EMB and PYZ. (B) Bacterial burden 48 h after antibiotic treatment. (C) Intracellular ROS levels in *M. tuberculosis* infected and antibiotic treated macrophages 48 h post infection (pi). (D-F) Macrophage activation after antibiotic treatment. Expression of (D) CD11b^+^ (E) CD80 and (F) MHCII on infected macrophages 48 h after antibiotic treatment. (G) Phosphorylation status of ERK1/2 inM. tuberculosisinfected macrophages after antibiotic treatment. (H) Expression of pro- and anti-inflammatory cytokines in *M. tuberculosis* infected and antibiotic treated macrophages 48 h pi. (I) Peritoneal macrophages isolated from C57/BL6 mice were infected with *M. tuberculosis* H37Rv at an MOI of 1:10 followed by treatment with 1 µg/mL of INH, RIF, EMB and PYZ for 48 h. Percentage of apoptotic cells after antibiotic treatment. Data are representative of three independent experiments (±SD). *P < 0.05, **P < 0.005, ***P < 0.0005.

Multiple antibiotic players are involved in treating active TB, thereby attempting to sterilize the host, and reduce the transmission risk and drug resistance (16). However, at the same time, these drugs are recommended for longer duration, thereby leaving after-effects and evolution of resistant strains (17). Macrophages are the primary immune cells that encounter *M. tuberculosis* and liberate a myriad of host defense-mediated toxic effectors such as reactive oxygen species (ROS), nitrogen oxide species (NOS), lysosomal enzymes, along with abundant proinflammatory cytokines and chemokines (18). Hence, we foremost investigated the impact of DOTS antibiotics: INH, RIF, EMB, and PYZ treatment on the proficiency of macrophages wherein we isolated peritoneal macrophages from C57BL/6 mice (Fig. 1A) and treated them with first-line antibiotics upon *M. tuberculosis* infection and evaluated the macrophages response. Expectedly, we observed that these anti-TB drugs displayed bactericidal activity and hence a significant reduction in bacterial burden within *M. tuberculosis* infected and DOTS-treated macrophages was seen (Fig. 1B). However, front-line drugs are never able to completely sterilize the host of *M. tuberculosis*. Therefore, we checked the influence of DOTS antibiotics on macrophage defense forces. We found that macrophages treated with INH, RIF, and EMB exhibited significant ROS generation to encounter the intracellular bacteria with moderate effect of PYZ in ROS formation (Fig. 1C). However, a significant reduction in the expression of CD11b^+^ (Fig. 1D) and co-stimulatory molecule CD80 (Fig. 1E) was observed in the antibiotic-treated macrophages. It is well established that CD4^+^ T helper cells are critical in containing *M. tuberculosis* pathogens that are induced by APCs with their cognate major histocompatibility complex class II (MHCII) molecule (19). Unfortunately, a significant depletion of MHCII expression was noted on macrophages treated with the frontline anti-TB drugs (Fig. 1F). Furthermore, antibiotics treatment ameliorated the extracellular signal-related kinase (ERK) pathway (Fig. 1G), which is crucial in generating a proinflammatory environment during infection (20) and is required for the protection against TB as well as for the induction of protective Th1 immune response. In addition to the ERK pathway, we explored the p38 signaling pathway responsible for triggering host protective immune responses during TB. However, we observed that DOTS treatment did not alter the p38 signaling pathway. Additionally, it became more evident through the q-PCR results (Fig. 1H) (primers listed in Table 1) that DOTS treatment downturned the expression of innate cytokines, autophagy, and macrophage activation-related genes. Interestingly, treatment with INH and RIF significantly decreased the apoptotic ability of *M. tuberculosis* infected macrophages (Fig. 1I), which is also one of the major defense strategies against intracellular pathogens. Altogether, these data suggest that DOTS therapy declined the initial innate immune response via downregulation of macrophage defense mechanisms, which is required to activate the adaptive immunity.

**TABLE 1:** Primers used in the study

Primer	Sequence (5′−3′)
TNF-alpha Forward Primer	TAGCCAGGAGGGAGAACAGA
TNF-alpha Reverse Primer	TTTTCTGGAGGGAGATGTGG
IL-10 Forward Primer	CATGGGTCTTGGGAAGAGAA
IL-10 Reverse Primer	AACTGGCCACAGTTTTCAGG
IL-6 Forward Primer	CCGGAGAGGAGACTTCACAG
IL-6 Reverse Primer	TCCACGATTTCCCAGAGAAC
IL-1 beta Forward Primer	CCCAAGCAATACCCAAAGAA
IL-1 beta Reverse Primer	GCTTGTGCTCTGCTTGTGAG
IL-22 Forward Primer	CCGAGGAGTCAGTGCTAAGG
IL-22 Reverse Primer	CATGTAGGGCTGGAACCTGT
IL-23 Forward Primer	AATAATGTGCCCCGTATCCA
IL-23 Reverse Primer	AGGCTCCCCTTTGAAGATGT
IL-12p40 Forward Primer	AAGGAACAGTGGGTGTCCAG
IL-12p40 Reverse Primer	GGAGACACCAGCAAAACGAT
Gapdh Forward Primer	AACTTTGGCATTGTGGAAGG
Gapdh Reverse Primer	GGATGCAGGGATGATGTTCT
ATG5 Forward Primer	ATGTCGTGTATGAAAGAAGCTGATG
ATG5 Reverse Primer	CTGGTCAAATCTGTCATTCTGCA
ATG7 Forward Primer	TTAATAGTGCCCTGGACGTTGGCTT
ATG7 Reverse Primer	CACCATTGTAGTAATAGCCCTTGAT
CD80 Forward Primer	ACCCCCAACATAACTGAGTCT
CD80 Reverse Primer	TTCCAACCAAGAGAAGCGAGG
CD86 Forward Primer	TGTTTCCGTGGAGACGCAAG
CD86 Reverse Primer	TTGAGCCTTTGTAAATGGGCA
MHCII Forward Primer	AGCCCCATCACTGTGGAGT
MHCII Reverse Primer	GATGCCGCTCAACATCTTGC

### DOTS treatment leads to AICD reducing the killing capacity of T cells

**FIG 2:**
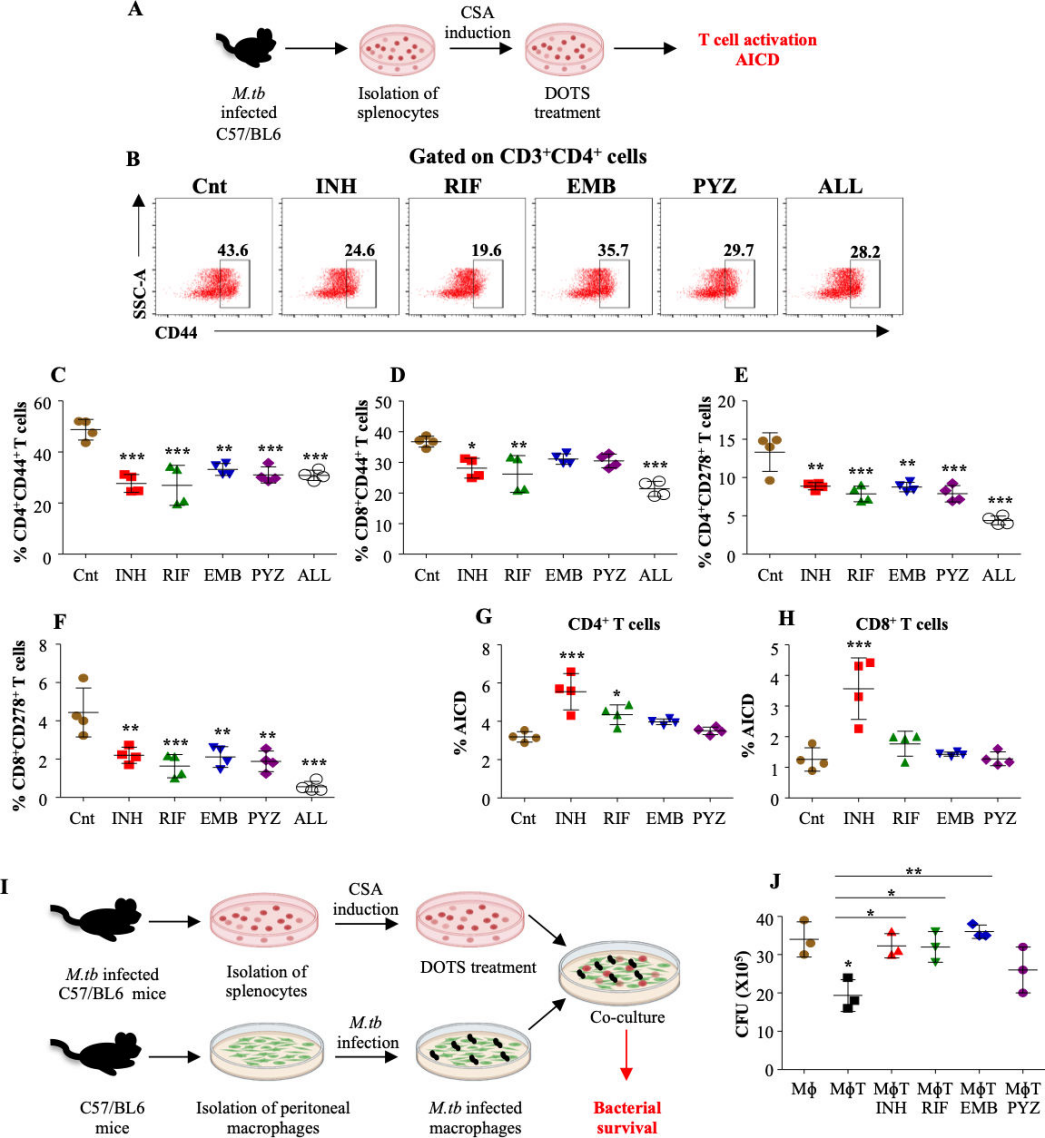
DOTS treatment reduces the anti-mycobacterial killing capacity of T cells. (A) Schematic representation of the T cell-DOTS model wherein splenocytes isolated from *M. tuberculosis* infected mice, stimulated with *M. tuberculosis* CSA were treated with DOTS antibiotics for 48 h followed by surface staining (see methods). (B) Representative dot plots and the percentage of CD44 expressing (C) CD4^+^ T cells and (D) CD8^+^ T cells after antibiotic treatment. Expression of CD278 on the surface of (E) CD4^+^ T cells and (F) CD8^+^ T cells after antibiotic treatment. AICD (CD95^+^CD69^+^) in the (G) CD4^+^ T cells and (H) CD8^+^ T cells following antibiotic treatment. (I) To determine the killing capacity of antibiotic-treated T cells, the *M. tuberculosis*-infected peritoneal macrophages were co-cultured with the ex-vivo stimulated *M. tuberculosis*-specific T cells pre-treated with the antibiotics followed by (J) CFU analysis 48 h pi. Data are representative of three independent experiments (±SD). *P < 0.05, **P < 0.005, ***P < 0.0005.

To better comprehend the impact of DOTS therapy on T cells, we performed an *ex vivo* experiment wherein C57BL/6 mice were infected with a low dose aerosol of *H37Rv* (~110 CFU) and splenocytes from infected mice were either left untreated (Control) or treated with individual drugs INH, RIF, EMB, and PYZ followed by immune profiling (Fig. 2A). For pathogen clearance, detectable T cell response is executed by T cell activation from resting T cells, which is crucial for cell division, upregulation of adhesion molecules, and generation of cytokines and cytotoxic peptides involved in the elimination of the pathogens (21). Interestingly, we found that activation of CD4^+^ T cells and CD8^+^ T cells in terms of the expression of CD44, which is a T cell activation marker, was significantly receded in both CD4^+^ T cells (Fig. 2B and C) and CD8^+^ T cells (Fig. 2D) upon DOTS treatment. Following the activation, for an effective immune response during TB, the T cells need to recognize its cognate antigen and proliferate further to mediate bacterial killing (22). Importantly, these proliferating T cells further differentiate into memory cells secreting effective cytokines and elevated T cell receptors, which is marked by CD278 /ICOS (inducible T-cell costimulator gene). It is a CD28-superfamily co-stimulatory molecule that is expressed on activated T cells and a co-stimulatory marker for T cell proliferation and cytokine production (23). Intriguingly, we observed a serious decline in CD278 expressed by CD4^+^ T cells and CD8^+^ T cells (Fig. 2E and F). Further, treatment of splenocytes with all four drugs together drastically reduced the CD4^+^ as well as CD8^+^ T cell activation indicating the possible harmful effects of long-term DOTS treatment (Fig. 2B through F). During AICD, cells undergo apoptosis as a mechanism to regulate the immune system, maintaining balance among cell division, differentiation, and cell death. Nevertheless, heightened apoptosis can lead to cell loss and impair the ability of stem cells to maintain homeostasis by replenishing new cells (24, 25). We also observed a significant increase in AICD in INH and RIF-treated CD4^+^ T cells (Fig. 2G) and CD8^+^ T cells (Fig. 2H) leading to T cell demise and mitigating the adaptive immune response in controlling infection.

To further understand the impact of DOTS treatment on T cell protective potential during TB, we co-cultured *M. tuberculosis*-infected peritoneal macrophages with drug-treated T cells isolated from *M. tuberculosis*-infected mice (Fig. 2I). It was startling to observe that, the drug regimen given subsided the killing capacity of T cells as there was no significant bacterial burden reduction in the macrophages co-cultured with treated T cells as compared to macrophage control (Fig. 2J) indicating the negative impact of anti-TB drugs on adaptive immunity

### DOTS dampens memory immune responses by targeting T-cell signaling pathways

**FIG 3:**
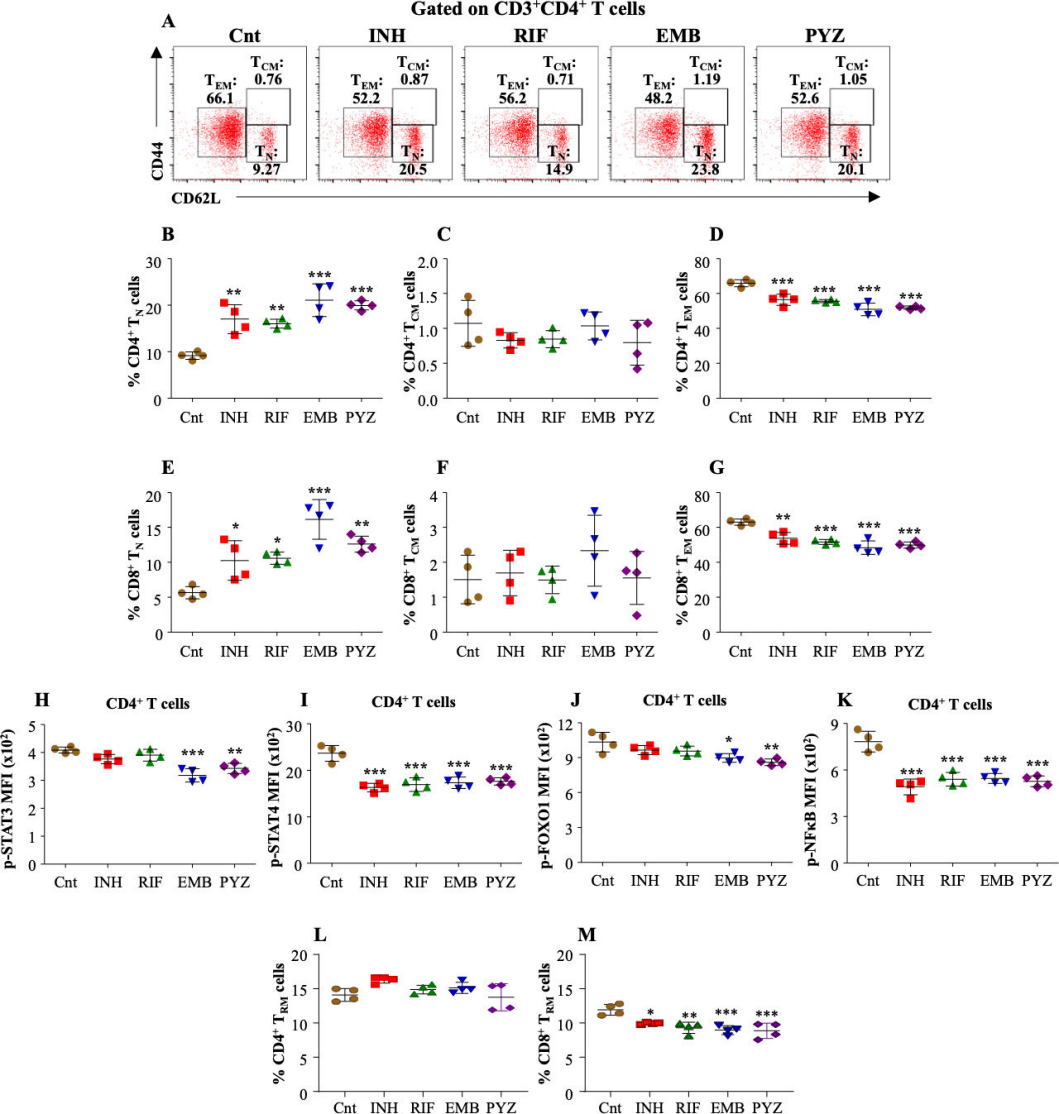
Antibiotic exposure restricts the differentiation of T_N_ cells into memory subsets. Splenocytes isolated from *M. tuberculosis* infected mice were *ex vivo* stimulated with *M. tuberculosis* CSA and treated with the antibiotics for 48 h followed by surface staining for memory cell profiling (see methods). (A) Representative dot plots and the percentage of (B) CD4^+^ T_N_ cells (CD62L^HI^CD44^LO^), (C) CD4^+^ T_CM_ cells (CD62L^HI^CD44^HI^) and (D) CD4^+^ T_EM_ cells (CD62L^LO^CD44^HI^) after antibiotic treatment. The percentage of (E) CD8^+^ T_N_ cells, (F) CD8^+^ T_CM_ cells and (G) CD8^+^ T_EM_ cells after antibiotic treatment. Phosphorylation status of transcription factors (H) STAT3, (I) STAT4, (J) FOXO1 and (K) NFκB in the antibiotic treated CD4^+^ T cells. The percentage of (L) CD4^+^ T_RM_ cells (CD62L^LO^CD44^HI^CD69^+^), (M) CD8^+^ T_RM_ cells after antibiotic treatment. Data are representative of three independent experiments (±SD). *P < 0.05, **P < 0.005, ***P < 0.0005.

The importance of differentiating naïve T cells into effective long-lived antigen-specific memory responses in protecting against TB infection has been known for decades. A pool of memory cells can mediate rapid reaction toward reinfection or reactivation of the disease (26). Amid drug-resistant TB, lengthy treatment is prescribed for more than 6 months; hence, it is vital to deduce whether, after such a long treatment, there is a possibility of disease relapse. Therefore, we investigated the naïve and memory population within CD4^+^ T cells and CD8^+^ T cells upon antibiotic treatment. Upon treatment of *M. tuberculosis* infected splenocytes with DOTS drugs, we observed a significant block in the differentiation of naïve CD4^+^ T cells (T_N_) into effector memory (T_EM_) and central memory (T_CM_) subtypes as the naïve population of CD4^+^ was significantly increased (Fig. 3A and B). With the minimal change in the T_CM_ population (Fig. 3C), there was a significant decrease in the CD4^+^ T_EM_ population in the DOTS-treated samples (Fig. 3D). A similar trend was observed in the CD8^+^ T cells (Fig. 3E through G). The transition of central memory to effector memory, together with their interchangeability, is crucial as the ratio between them plays a pivotal role in inducing long-term effects. However, a decrease in effector memory with a concomitant increase in naïve cells indicated that the DOTS treatment inhibited T naïve differentiation into memory subtypes, which may lead to compromised immunity.

STAT3 is the transcription factor that is majorly involved in T cell activation, differentiation, and generation of memory responses. Moreover, during *M. tuberculosis* infection, it is known to stimulate Th17 and activate cytokines aimed at containing the pathogen (27). We observed that EMB and PYZ significantly lowered the expression of pSTAT3 within CD4^+^ T cells (Fig. 3H). STAT4 is the transcription factor that is activated in response to IL-12 and IFNγ and stimulates Th1-mediated host protective responses during the infection (28). It was observed that in CD4^+^ T cells, each drug reduced the expression of pSTAT4 (Fig. 3I). It is established that inhibition of the Akt signaling pathway enhances FOXO1 expression within the nucleus and results in the formation of memory cells involving STAT signaling pathways as well (29). Thereafter, we also analyzed the regulation of pFOXO1 upon DOTS therapy, which resulted in its significant decrease in CD4^+^ T cells during EMB and PYZ treatment (Fig. 3J). We also examined pNFκB expression, which is being identified as one of the important transcription factors in reducing bacterial load and the granuloma size (30). Every individual drug of the DOTS regimen declined its expression levels in CD4^+^ T cells (Fig. 3K). We further observed a marked decrease in the percentage of resident memory CD8^+^ T cells after DOTS treatment with no effect on CD4^+^ T cells (Fig. 3L and M). However, we did not observe any significant difference in the cytokine levels after DOTS treatment. Overall, DOTS treatment reduces the generation of an optimal memory response, which is a definite cause of disease relapse.

### DOTS treatment makes the host vulnerable to disease reactivation

**FIG 4:**
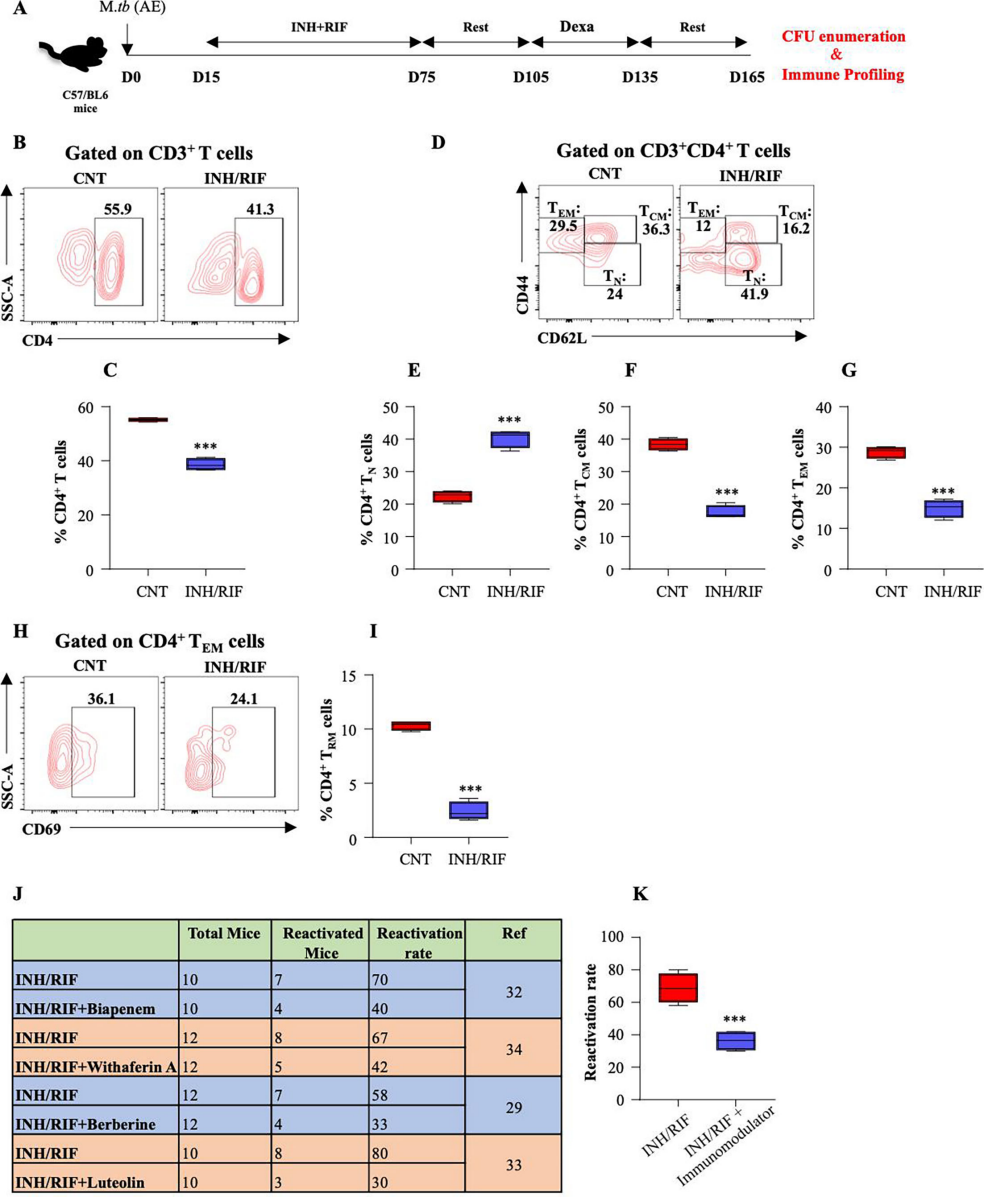
INH/RIF therapy reduces the expansion of central memory T cells thereby inducing the host vulnerability of TB reactivation. (A) Schematic representation of the reactivation model used in the study. C57BL/6 mice were infected with *M. tuberculosis* through low dose aerosol infection model and treated with INH/RIF for 60 days followed by dexamethasone (Dexam) treatment for immune suppression. At day 165 post infection, the mice were sacrificed for CFU and immune profiling. (B and C) Percentage of CD4^+^ T cells in the lungs of *M. tuberculosis* infected and INH/RIF treated mice. (D) Dot Plot showing the profile of memory CD4^+^ T cells. Percentage population of (E) Naïve (T_N_) CD4^+^ T cells, (F) Central memory (T_CM_), and (G) Effector memory (T_EM_) CD4^+^ T cells in the lungs of *M. tuberculosis* infected and INH/RIF treated mice. (H and I) Percentage population of resident memory (T_RM_) CD4^+^ T cells in the lungs of *M. tuberculosis* infected and DOTS treated mice. (J) Table showing the reactivation rate in case of adjunct formulation with different phytochemicals along with INH/RIF therapy. (K) Cumulative rate of reactivation in case of adjunct formulation with INH/RIF therapy. Data in panels B–I are representative of three independent experiments (±SD). *P < 0.05, **P < 0.005, ***P < 0.0005. Data in panels J and K are adapted from references 29, 32–34.

Although we are well versed in the countereffects of DOTS therapy, however, the deep-rooted consequences on host immunity are still uncovered. On that account, we further authenticated the *ex vivo* outcomes in mice models while giving them long-term drug treatment wherein *M. tuberculosis* infected and INH/RIF treated mice were subjected to the reactivation by dexamethasone (Dexam) treatment followed by immune profiling and CFU analysis (Fig. 4A). No detectable CFU was observed in the lungs of drug-treated animals indicating host sterilization. When we profiled the immune cells in the lungs, we observed a significant reduction in the number of CD4^+^ T cells in the INH/RIF-treated animals (Fig. 4B and C). Furthermore, there was a significant halt in the differentiation of T_N_ cells toward memory subsets in line with the *ex vivo* results (Fig. 4D through G). Interestingly, resident memory T cells (T_RM_), which play a pivotal role during secondary infections (31), were significantly lower in the lung of INH/RIF treated mice (Fig. 4H and I). These data strongly suggest that the anti-TB therapy deprives the host of long-term memory responses making it vulnerable to disease recurrence. In line with this, we have previously shown how INH/RIF treated animals are more prone to disease reactivation while if accompanied by an immunomodulator, the reactivation rate significantly decreases (Fig. 4J and K) (29, 32–34).

## DISCUSSION

Conventional DOTS therapy functions by interacting with the host immune system and leveraging its effects to successfully eradicate a pathogen from the body. Besides its effectiveness in treating TB, there are significant implications associated with the therapy (35). Although DOTS therapy remains a fundamental component of TB treatment, there is a lag in understanding its immediate and prolonged effects on the host immune system.

Following the established literature, we demonstrated the circumstantial influence of anti-TB therapy on the innate arm of immunity, which is known to have a primary role in responding to infection. Mononuclear phagocytic cells seize the pathogen by initiating signaling cascades and prompting the macrophage activation to restrain bacterial proliferation. Nonetheless, DOTS treatment, achieved the anticipated reduction in bacterial growth. However, we have shown that it concurrently impedes macrophage activation by diminishing the expression of key markers such as CD11b^+^, CD80, and MHCII (Fig. 1). Collectively, these markers are present on activated macrophage subsets, guiding the polarization of macrophages to M1 phenotype, and this facilitates the processing of antigens to T cells, playing a crucial role in triggering host-specific adaptive immune responses (36). DOTS treatment additionally diminishes the levels of innate cytokines by decreasing ERK activation and subsequently suppresses apoptosis and autophagy, a foundational mechanism employed by macrophages to counteract the pathogen evading immune system (37) (Fig. 1).

The initial phase of T cell activation in the early stages of *M. tuberculosis* infection plays a pivotal role in determining the fate of the disease. Moreover, this is largely reliant on CD4^+^ T cells, which trigger the release of potent cytokines to combat the infection (26). Hence, we investigated the early signal transduction events occurring in CD4^+^ T cells during DOTS treatment upon *M. tuberculosis* infection. We observed a concomitant reduction in the early T cell activation markers (Fig. 2). Moreover, our observations revealed a noteworthy prevalence of AICD within both CD4^+^ and CD8^+^ T cells during the DOTS treatment. Moreover, these drugs were also found to diminish the cytotoxic capabilities of DOTS-treated T cells in the co-culture settings (Fig. 2).

**FIG 5:**
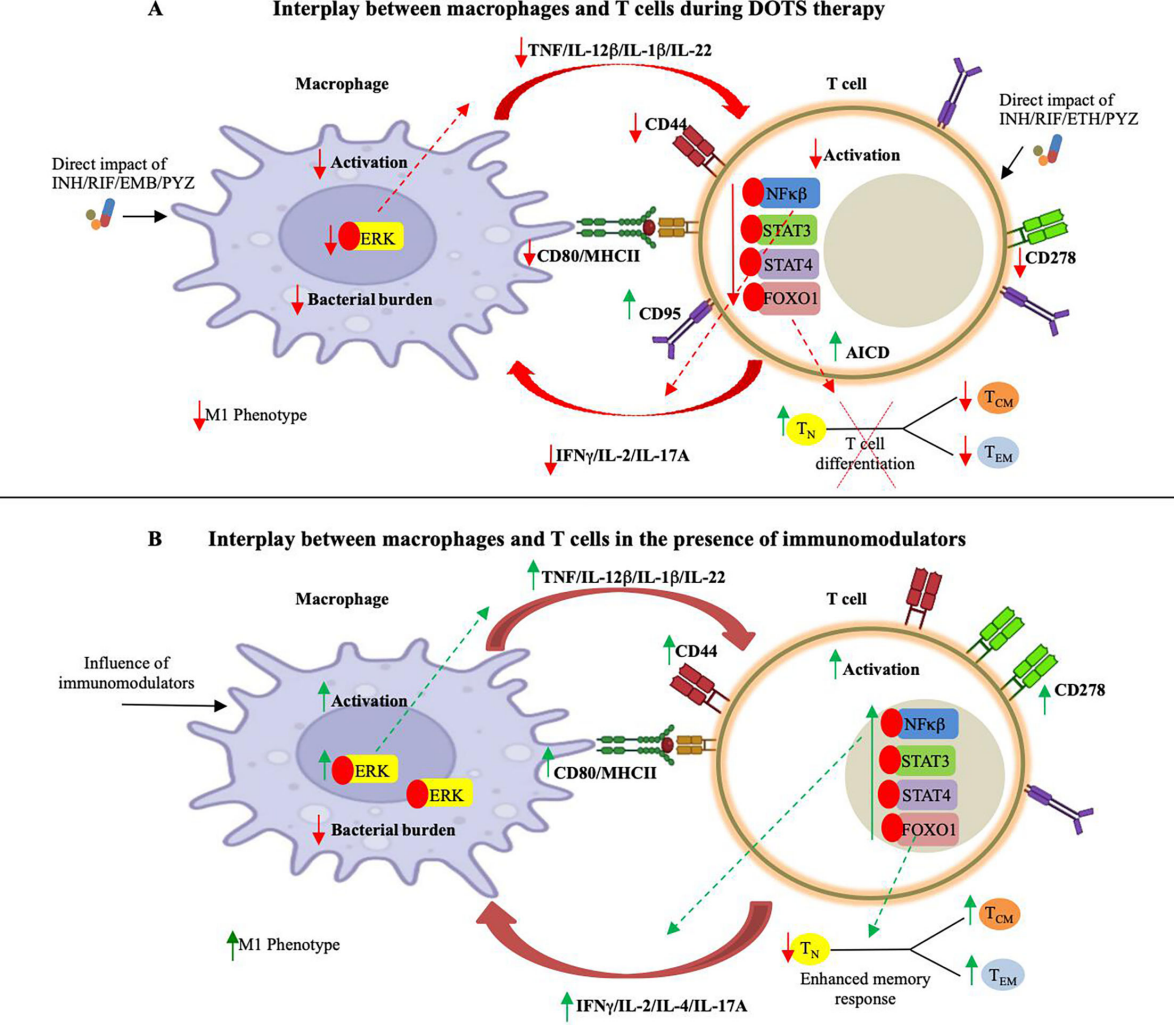
Consequences of DOTS therapy and immunomodulators on host immunity upon TB infection. (A) In response to *M. tuberculosis* infection, DOTS treatment dissuades the macrophage and T cell activation by downregulating key transcription factors that further suppress the proinflammatory cytokines response and memory generation. (B) In contrast, the incorporation of immunomodulators enhances the interaction within the innate and adaptive immune populations by actively eliciting inflammatory responses and further upregulating the effector and memory functions of T cells leading to reduced TB recurrence.

Differentiation of naïve T-cells into antigen-specific T-cell subsets is crucial in providing immunity against *M. tuberculosis*. CD4^+^ T cells stimulate proinflammatory cytokines IFNγ and IL17, thereby enhancing Th1/Th17 mediated immune response (38). Upon T-cell activation, when CD4^+^ T cells or CD8^+^ T cells are differentiated into their respective subsets, the related transcription factors are stimulated and lead to the expression of particular cytokines (39). Upon administering DOTS to infected splenocytes, we observed a significant reduction in the activation of key transcription factors such as STAT3, STAT4, NFΚβ, and FOXO1. Apart from creating a pro-inflammatory environment, these factors also play a crucial role in accelerating memory responses (Fig. 3). In uniformity to the above results, we observed a concomitant reduction in the memory cells of T cell subsets that are pivotal in preventing the disease relapse or recurrence (Fig. 3). These results were also replicated in the *in vivo* settings wherein DOTS therapy tremendously reduced the memory pool of the host making it highly vulnerable to disease reactivation (Fig. 4). Our investigation has illuminated the comprehensive immunological aspects of DOTS therapy, which serves as a keystone in TB treatment. Moreover, the occurrence of adverse side effects can contribute to patient non-compliance, potentially leading to the emergence of resistant strains and disease reinfection and reactivation. Consequently, there is a pressing need for a multifaceted approach, involving the complementation of DOTS with immunomodulatory drugs to strengthen the TB control efforts and overcome the challenges raised in treating TB (Fig. 5). A range of existing immunomodulators are available that can amplify the immune responses and facilitate the establishment of persistent memory responses (40, 41). Our previous findings have identified several such key immunomodulators such as Bergenin, allicin, Gingerol, Berberine, Withaferin A, and Biapenem. Although every immunomodulator harbors distinct mechanisms to accelerate host immunity, in general, these compounds have been known to play a crucial role in the battle against TB. These immunomodulators promote the expansion of memory cells and encourage pro-inflammatory responses within the innate and adaptive immune systems. Moreover, they also activate distinct signaling pathways that contribute to a more robust immune defense against the infection (29, 32, 34, 42–44). For instance, Biapenem was shown to modulate p38 signaling in host macrophages while Withaferin-A and Berberine induced STAT pathway and NOTCH-Akt axis, respectively, in the T cells to induce host protective memory immune responses (29, 32, 34). We have also shown that the integration of host-targeted inhibitors such as AGK2 (SIRT2 inhibitor) constrains intracellular growth of *M. tuberculosis* and also improves vaccine efficacy by influencing host immunity (45, 46).

Given the well-established knowledge that administering antibiotics can result in unavoidable adverse effects and their misuse can contribute to drug resistance, it becomes essential to comprehend the subsequent impacts of each medication. This understanding aids in bolstering the host immune response, which may be compromised, particularly by these medications. In conclusion, novel formulations having immunomodulatory potential are the need of the hour to compensate for the immune inhibitory effects of ATT. These formulations also have immune potentiating effects that can reduce the host vulnerability of TB recurrence, which is the major challenge being faced globally (Fig. 5).

## MATERIALS AND METHODS

### Mice

C57BL/6 mice (6–8 weeks old) weighing 20–25 g were preserved and nourished in sterile conditions at the animal facility of ICGEB, New Delhi, India. The mice were further procured as per the experimental requirements.

### Bacterial strain

Mycobacterial stocks were cryopreserved in 20% glycerol (Sigma) and were kept at −80°C. Further, the exponential culture of H37Rv was maintained in 7H9 (Middlebrook, Difco) medium supplemented with 10% oleic acid, albumin, dextrose, and catalase (OADC; Difco), 0.05% Tween 80%, and 0.2% glycerol for experimental purpose.

### Isolation of mouse peritoneal macrophages

2 mL of sterile thioglycolate (Brewer modified, BBL, BD Biosciences: 4% [wt/vol] in water) was given intraperitoneally to C57/BL6 mice aged 6–8 weeks 5 days before harvesting of macrophages. Subsequently, macrophages were isolated from peritoneum lavage using ice-cold sterile phosphate-buffered saline (PBS) and counted using a hemocytometer chamber to seed the cells. The cells were further incubated overnight in RPMI-1640 medium supplemented with 10% fetal bovine serum (Thermofisher Scientific Inc or Hyclone) at 37°C and 5% CO_2_. The non-adherent cells were washed using sterile 1× PBS and an adherent monolayer of macrophages was infected with the mycobacterial strain with MOI 1:1.

### *Ex vivo* macrophage infection

Mid-log phase cultures were maintained from bacterial cryo-stocks, and single-cell suspension was prepared to infect the cultured macrophages. 4 h post-infection, the extracellular bacteria were washed off twice with sterile 1× PBS, and the cells were further treated with INH (1 µg/mL), RIF (1 µg/mL), EMB (10 µg/mL) and PYZ (10 µg/mL).

### *Ex vivo* splenocytes experiment

Single cell suspension of splenocytes was extracted from the spleen of *M. tuberculosis* infected mice using sterile frosted slides in 1× PBS. Eventually, different concentrations of drugs were administered to cells for protein, RNA, and immune cell profiling experiments.

### *M. tuberculosis* infection in mice and CFU determination

Axenic cultures of drug-sensitive strains were maintained and sonicated to prepare single cell suspension and give infection using Madison aerosol chamber (University of Wisconsin, Madison, WI) with its nebulizer pre-adjusted to deliver approximately 110 CFUs to the lungs of mice. The lungs of mice were further isolated and homogenized in sterile PBS and were plated onto 7H11 Middlebrooks (Difco) plates containing 0.05% Tween-80, 10% OADC (Difco). Different dilutions of homogenates were plated and incubated at 37°C for 21–28 days. *M. tuberculosis* colonies were counted, and CFU were enumerated at various time points.

### Apoptosis and CellROX assay

Peritoneal macrophages were infected with *M. tuberculosis* strains at a 1:10 MOI. After 4 h post-infection, cells were washed with 1× PBS to eliminate extracellular bacteria. Treatment with different DOTS drugs was then administered, followed by incubation at 37°C for various time points. Apoptosis was assessed by staining cells with annexin V/7AAD (Biolegend) according to the manufacturer’s instructions. Additionally, intracellular ROS in *M. tuberculosis*-infected peritoneal macrophages following treatment for 24 h was detected using CellROX (ThermoFisher Scientific).

### Drug administration

For DOTS therapy, 100 mg/L of INH and 60 mg/L RIF were administered to mice of treated groups in drinking water, which was changed every alternative day. For immune suppression, 5 mg/kg of dexamethasone was given intraperitoneally, thrice a week for 30 days.

### RNA isolation and qPCR

Peritoneal macrophages and splenocytes were subjected to standard RNA isolation protocol to extract total RNA, followed by cDNA synthesis utilizing the iScript cDNA synthesis kit from Bio-Rad. Real-time PCR analysis was conducted using SYBR Green (SG) Master Mix from Bio-Rad on a Bio-Rad Real-Time thermal cycler (BioRad, USA) for quantitative RT-PCR analysis. Details regarding the primers utilized in this study are provided in Table 1.

### Protein isolation and immunoblotting

The cultured cells were lysed with radioimmunoprecipitation assay (RIPA) buffer (50 mM Tris, pH 8.0, 150 mM NaCl, 1.0% NP-40, 0.5% sodium deoxycholate, 0.1% SDS) supplemented with 1× protease and phosphatase inhibitor cocktail (Thermo Scientific) to prepare whole cell lysate.

### Immune profiling by flow cytometry

Single-cell suspension was prepared from the lungs of mice belonging to different groups using frosted slides in ice-cold RPMI 1640 (Hyclone) supplemented with 10% fetal bovine serum (FBS). (Red blood cell (RBC) lysis buffer was used to lyse RBCs, and cells were washed with 10% RPMI 1640. Cells seeded with the density of 1 × 10^6^ cells were seeded in 12 wells for staining post-cell count. Cells were stimulated with complete soluble antigen (CSA) of H37Rv overnight. After surface staining, cells were fixed with 100 µL fixation buffer (BioLegend) for 30 min. Subsequently, for intracellular staining of cytokines, 0.5 µg/mL brefeldin A and 0.5 µg/mL of Monensin solution (BioLegend) were added during the last 4 h of culture, and then the cells were then washed twice with fluorescence-activated cell sorting (FACS) buffer (PBS + 3% FBS). The cells were permeabilized using 1× permeabilizing buffer (BioLegend) and then stained with fluorescently labelled anti-cytokine antibodies. The fluorochromes intensity was assessed by flow cytometry (BD LSRFortessa Cell Analyzer-Flow Cytometers, BD Biosciences) followed by data analysis via FlowJo (Tree Star, USA).

### Antibodies

The following antibodies were used for this study: anti-mouse: CD3-Pacific Blue, CD4-PerCPCy5.5, CD8-APCCy7, CD44-FITC, CD62L-APC, IFNγ-APC, IFNγ-BV510, IL17-PECy7, IL17-BV650, and tumor necrosis factor alpha (TNFα) from Biolegend, USA; ERK, p-ERK, and β-actin from Cell Signaling Technology.

### Statistical analysis

Graph pad Prism software was used for all experimental procedures. Significant differences among the groups were indicative of a two-tailed unpaired Student’s *t*-test or one-way analysis of variance (ANOVA). In the figures, significant variations are demonstrated as follows: *P < 0.05; **P < 0.005, and ***P < 0.0005.
